# Application of conjugated materials in sports training

**DOI:** 10.3389/fchem.2023.1275448

**Published:** 2023-09-27

**Authors:** Kun Zhu, Longfei Zhao

**Affiliations:** ^1^ Graduate School, St. Paul University, Tuguegarao, Philippines; ^2^ Sports and Health College, Guizhou Medical University, Guiyang, China

**Keywords:** physical activity training, conjugated materials, sensing technology, heart rate monitoring, sports equipment design, new chemical materials

## Abstract

In recent years, with the rapid development of the sports industry, the quality of sports training products on the market is uneven. Problems such as inaccurate detection of athletes’ physical indicators, low comfort of sportswear, and reduced satisfaction with sports equipment often occur. To this end, this article proposes to apply conjugated materials with excellent optical, electrical, thermal and other properties to sports training and sports products, by summarizing the properties of conjugated materials and their applications in sports training, explores the potential of conjugated materials in improving athletes’ training effects, monitoring sports status, and improving sports equipment. This article rates the application of conjugated materials in sports training products in terms of comfort, waterproofness, portability, lightness, aesthetics and breathability. The results showed that the average scores of the 20 sports participants on sportswear were 9.0475, 9.0075, 9.01, 9.025, 9.0325 and 9.04 respectively; the average scores on sports shoes were 9.035, 9.055, 9.02, 9.085, 9.0175 and 8.9975 respectively. Research shows that applying conjugated materials to sports training can improve athletes’ performance and contribute to the better development of sports.

## 1 Introduction

Conjugated materials are materials with better performance and stronger efficacy due to the development of science and technology, which are used in all areas of life. It has also been widely used in sports training equipment and sports training equipment. These applications can improve the safety and comfort of sports, which is very important for sports training refueling, highlighting the necessity of using conjugated materials in sports training. With the development of sports training, there are some problems in the use of traditional materials in sports training, such as low application rate in sports training, insufficient and advanced research and development content, and high production cost of sports training equipment and equipment made from it. In order to solve these problems, this paper takes conjugation as the core of discussion, and makes clear the application status of conjugation materials in sports training. Secondly, this paper discusses the importance of conjugated materials in sports training, and then introduces the application of conjugated materials in sports training from different aspects to promote the more rational and perfect application of conjugated materials in sports training.

Different materials have different effects on sports training. The application of different materials in sports training equipment and equipment can improve the safety, comfort and sports performance of sports, which is of great significance for sports training. Hassabo, Ahmed G believed that nanotechnology has made great progress in textile materials. He applied it to sports training clothing and strengthened the research on the use of nanotechnology in the manufacture of sports clothing (Hassabo et al., 2023). Huang Ke introduced the advantages and main technologies of carbon materials used in sports equipment at the present stage. He analyzed the application of carbon materials in sports equipment and believed that it had a certain impact on improving sports performance (Huang, 2019). Wan Xupeng believed that plastics and their composite materials have the characteristics of lightweight. By introducing its application in sports products, he further explained the advantages of plastics and their composites in sports equipment (Wan and Niu, 2019). Li Jiaqi introduced the application of plastic composite materials in sports facilities and fitness equipment. He analyzed the advantages of plastic composite materials in the field of sports facilities and fitness equipment, and prospected the future prospects of plastic composite materials (Li, 2020). In general, although the use of different materials in sports clothing and equipment has brought many benefits, there are still problems in the water resistance and breathability of clothing, and there are also shortcomings in the accuracy of the equipment produced for the monitoring of athletes’ physical indicators. Conjugated materials have advantages in monitoring and waterproofing, which can effectively solve these problems.

For the water resistance of conjugated materials, as well as the monitoring of conjugated materials in body indicators, many experts and scholars have carried out in-depth research, and achieved some research results. Zhou, Lingyun studied the properties of conjugated materials such as high brightness, low cytotoxicity and fluid stability. He found that relying on these properties can promote the development of chemical sensors, fluorescence imaging, and disease diagnosis, and these developments can improve the accuracy of monitoring physical indicators (Zhou et al., 2019). Tian, Li studied N-type conjugated polymer materials containing fluorine and amino side chains. He found that it had excellent hydrophobic properties, and used it in solar batteries to greatly improve their water resistance (Tian et al., 2019). Meng, Qing-Wei designed a series of super-hydrophilic aromatic sulfonate materials through the study of conjugated materials, including the continuous addition of conjugated systems and the development of chlorine-resistant films through chemical modification of ionizable groups. The results show that the combination of large conjugated systems and ionizable groups is the key to the injection of membranes with excellent chlorine resistance and water permeability, and the conjugated materials have strong water resistance (Meng and Ge, 2020). In summary, conjugated materials have great advantages in water resistance and body index monitoring. If it is applied to sports training clothing and equipment, it should be able to effectively solve the problems of poor water resistance and inaccurate monitoring of body indicators existing in existing sports clothing.

Traditional materials have limited functionality in sports training, lack adaptability, low durability and sustainability, and lack intelligence. They can only meet certain specific needs and cannot provide a wider range of functions. Conjugated materials, on the other hand, exhibit many advantages in performance compared to other traditional materials. Conjugated materials can combine materials with different properties to achieve better performance and functionality. For example, intelligent wearable devices and equipment can use conjugated materials to provide real-time data and feedback, helping athletes better understand their training status and adjust their training plans. In addition, conjugated materials can provide better adaptability, durability, and sustainability to meet the needs of athletes and reduce their impact on the environment. The result of these advantages is that sports equipment and clothing have many functions, such as helping to improve the safety of athletes and sports enthusiasts during sports, and reducing muscle injuries caused by sports equipment. Of course, in addition to the above advantages, the plasticity of conjugated materials is very strong. When manufacturing sports equipment, it can be adjusted according to the characteristics of the athlete’s body and athletes to create a more comfortable experience for sports personnel. At the same time, the development of competitive sports makes the equipment requirements in sports training more high, conjugated materials have very excellent performance. It can be applied to sports training, which can motivate athletes to achieve better results, and then promote the faster and stronger development of various sports.

## 2 Potential application of conjugated materials in sports training

Conjugated materials are a class of polymer materials with special structures, possessing special electronic structures and optical properties. Firstly, π electrons in conjugated materials can quickly conduct electrons, making them highly conductive. This makes conjugated materials suitable for manufacturing flexible sensors, smart textiles, etc. In motion training, for monitoring motion status, recording motion data, etc. Secondly, conjugated materials can absorb, emit, and conduct light from electronic energy levels, exhibiting rich optical properties. Therefore, conjugated materials can be applied to photosensitive sensors, fluorescent materials, etc. In sports training, for real-time monitoring and evaluation of body posture, motion amplitude, etc. In addition, conjugated materials have good flexibility and plasticity, which can come into contact with the surface of the human body and adapt to the shape and curves of body movement. Therefore, conjugated materials can be used to manufacture fitted sensors, wearable devices, etc., providing a comfortable wearing experience and highly accurate motion data collection in sports training (Liu et al., 2021; Yin et al., 2021). Then, conjugated materials can achieve selective adsorption and transport of specific molecules or ions by adjusting the molecular structure of the material. This enables conjugated materials to be applied in biosensors, drug delivery systems, etc. In sports training, for detecting specific indicators and achieving targeted therapies. Finally, conjugated materials have high chemical stability and durability, and can maintain performance stability in complex environments and long-term use. This enables conjugated materials to be reliably used under various conditions in sports training, such as humid environments, high temperature environments, frequent movement changes, etc. By using conjugated materials, higher levels of monitoring and assistance can be provided for sports training, meeting the needs of people engaged in sports for data recording, exercise evaluation, wearable devices, and more. At the same time, the development of conjugated materials in fields such as sports science and biomedical engineering has also provided new possibilities for improving sports training methods and improving sports effectiveness.

Conjugated materials can often perform safety functions in the production of sports equipment. Because the main function of various protective articles, clothes and different protective pads made of conjugated materials is to protect the health of athletes and avoid injuries during sports (Shi et al., 2023; Tian et al., 2023). In this case, all the load is concentrated on the upper half of the body, which can lead to fatal neck injuries. No matter what kind of movement, conjugated materials can be said that good energy absorption and effective damping are the most important value of materials used in the production of sports equipment. In addition to requiring a lighter load on the user in a collision, athletic clothing should also be comfortable and should not restrict the wearer’s movement. Adequate breathability is also important for maintaining a high level of athletic performance over time, and it determines the ability of clothing to allow moisture to pass through the diffusion process. Clothing can be made of conjugated materials, because open-hole foam and blind holes improve the ability of steam transmission, help athletes in sports is good breathability of clothing, protect good athletes.

**FIGURE 1:**
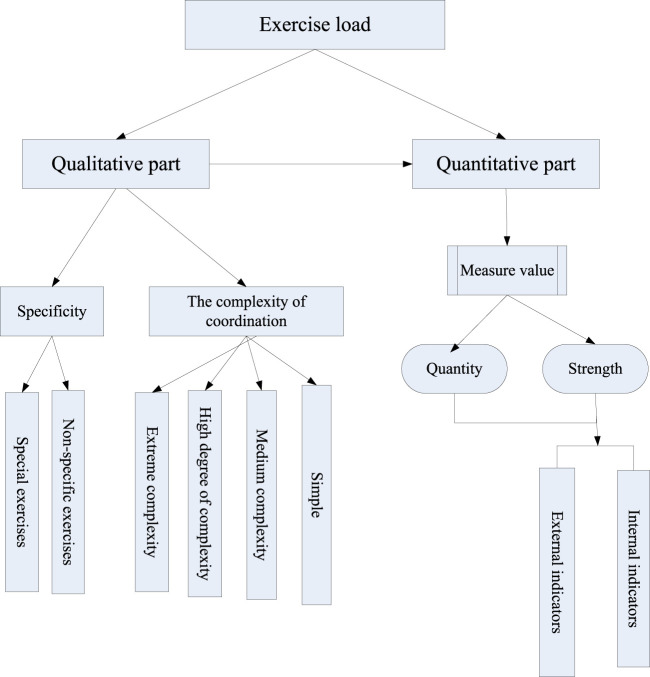
Exercise load introduction.

There should be appropriate demand and quantity of physical exercise in the training process (Appelbaum and Graham, 2018; Kasper, 2019; Robert, 2021). Qualitative is the basis of quantitative, and qualitative facts would determine the real effect of the training means and methods adopted. Only when the quality of training means and methods is analyzed and then quantified, can the load be accurately measured. As can be seen from Figure 1, proper exercises in training should have the necessary equipment. The sport is specific, which means that the load must meet the requirements of the competition in which the athlete participates and follow their own training level. Therefore, the exercise load in training can be divided into special exercises and non-special exercises. Specific practice is a direct factor to improve the performance of specific sports, non-specific practice is an indirect factor.

**FIGURE 2:**
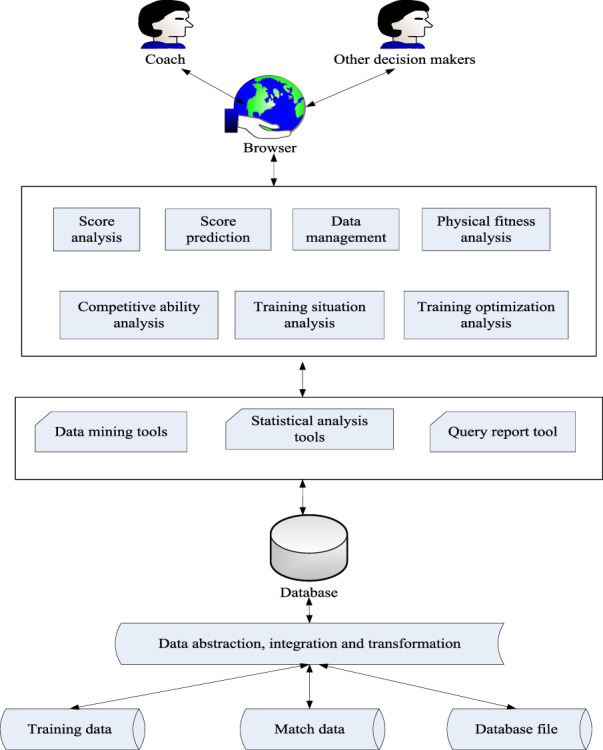
Module diagram of sports training data analysis.

In sports training, it is often necessary to analyze and make decisions on various training and competition data to improve the scientific level of sports training and the performance of athletes. Scientific analysis and decision-making not only need a large number of reliable data as the basis, but also need to be convenient and reasonable data organization as a guarantee to meet the different levels of decision-making needs of different levels of personnel. Data acquisition is responsible for transferring existing data to a database. The system’s data includes current training and competition information for each team or event, as well as archived data from existing applications. The data extraction, integration, and transformation module is responsible for extracting data related to database elements from data sources. According to the design of the database, the motion data can be changed through synthesis, reconstruction, screening and transformation to generate unified analysis data. The module diagram of sports training data analysis is shown in Figure 2.

## 3 Application of conjugated materials in motion state monitoring

### 3.1 Application of optical fiber sensing technology in sports training

Sensing technology is a multidisciplinary subject with both theory and practice in today’s society (Yousefi et al., 2019; Tyagi et al., 2020). Sensor technology is the use of various devices to detect information technology (Cao et al., 2019; Lv et al., 2022). Sensor technology plays a very important role in information processing system. It is one of the three constituent units of information processing systems (sensors, communication systems, computers) that have entered many production, research, and life. Sensor technology includes precise detail and high-performance development and assembly technology, and it also includes sensor soft technology on how to use sensor devices in various ways (Gu et al., 2021). According to the function of the sensor, it can be divided into physical sensors, chemical sensors and biological sensors. Sensors are classified according to measurement quantity as shown in Table 1.

**TABLE 1:** Classification by measurement.

Classification	Measurement	Sensor type
Physical sensor	Mechanical mass	Pressure sensor, force sensor, torque sensor, speed sensor, acceleration sensor
	Thermal energy	Temperature sensor, heat flow sensor, heat conduction sensor
	Optical quantity	Infrared light sensor, ultraviolet light sensor, brightness sensor, chromaticity sensor
	Magnetic quantity	Collision field strength transmitter, magnetic flux sensor
	Electrical quantity	Current sensor, voltage sensor, electric field strength sensor
	Acoustic volume	Noise sensor, ultrasonic transmitter, surface acoustic wave sensor
	Ray	X-ray sensor, Y-ray transmitter
Chemical sensor	Gas sensor, humidity sensor	
Biosensor	Biochemical quantity	Immune blood type transmitter, microbial type sensor, blood gas sensor
	Physiological quantity	Pulse sensor, heart tone sensor, body temperature sensor, blood flow sensor, breathing sensor

**FIGURE 3:**
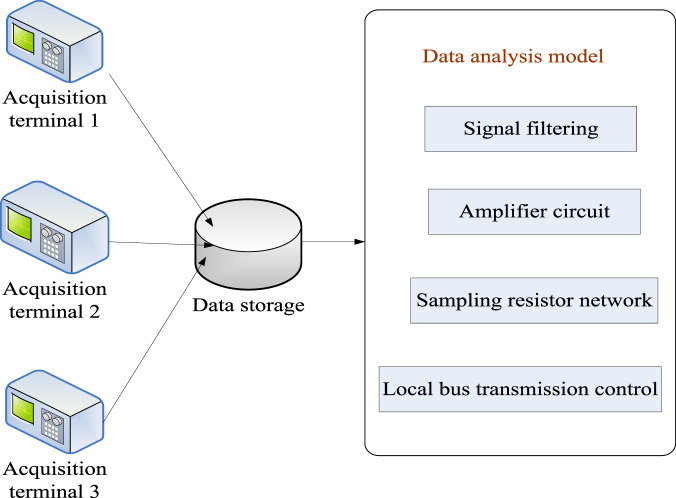
Sports load status monitoring data acquisition module.

As people pay more attention to the health of the body, they begin to gradually increase the intensity of physical training. For non-professional athletes, sometimes it is not possible to handle exercise intensity correctly. Therefore, it is necessary to study a technology to effectively monitor the load intensity of sports training, and the use of sensor technology can effectively monitor the exercise training of various sports players. This can help to realize the condition monitoring and optimal control of sports training. By combing with the analysis results of the characteristic parameters of the sports load state, it realizes the optimal monitoring results of the sports state. The careful design of the characteristics of the high load state of exercise can inform the state of care and management of exercise, which is important to support sports training and healthcare. The sports load status monitoring data acquisition module is integrated into Figure 3.

Accurate and timely exercise monitoring is an important prerequisite for scientific exercise training. In order to meet the physical needs of the competition, it is necessary to have some accurate and objective measurement methods in sports training to make the intensity and weight of the exercise close to or beyond the limit of the body, and to determine whether the athlete’s physical condition is suitable for the next training, to prevent overtraining. Physical indicators, psychological indicators, biochemical indicators and other functional indicators are very important, and the indicators selected by different sports are often different. Biosensors have many characteristics (such as sensitivity and accuracy, easy to operate, cheap, easy to mass production, can be online *in vivo* monitoring and other features) and have been applied in many fields, especially in the field of sports training. They can monitor the blood sugar, blood lactic acid, hemoglobin and myoglobin of athletes during sports.

### 3.2 Combination of sensing technology and conjugated materials in motion state monitoring

Sports training monitoring refers to the monitoring and control of athletes’ training, physical activity, fitness, daily nutrition, competitive performance and other aspects within a period of time (Suchomel et al., 2021; Xing et al., 2023). The coach or the athlete can adjust the training according to the monitoring. It includes training amount, training intensity, training density, training frequency and training duration. The monitoring of these indicators also tests whether the training program is reasonable and effective to a certain extent.

Sensing technology and conjugated materials are commonly used in sports training, measurement of athletes’ body dimensions, and physical and physiological studies. In the sports training, the coach through the analysis of the game to determine the characteristics of the athletes, but also can use this method to understand the characteristics of the opposing athletes. Sensing technology and conjugated materials play an increasingly important role in various stages of sports training. At the level of athlete selection, sensor technology can be used to collect athletes’ personal information, such as name, address and other basic information, as well as body parameters such as height and weight. It can be used as a reference factor for future coaches to choose the direction of sports training or to plan their sports training. At the level of sports training, various performance data of each athlete in different training links are collected through monitoring the training process (such as physiological and biochemical indicators, special indicators, prediction and evaluation of athletes’ special results, diagnosis of training states such as physical function, technical tactics and psychological state). Physiological and biochemical indicators are directly measured by the instrument, which can record and analyze physiological and biochemical parameters during exercise. Athletes’ physical activity monitoring data often include heart rate, blood pressure, blood and other indicators, monitoring and recording athletes’ sports and training content, and finally data analysis. In daily training, the athlete’s diet is still strictly controlled and recorded, especially in the weight requirements of the more obvious sports. For example, in the monitoring of weightlifting, free combat and other events, the software evaluates these comprehensive indicators to obtain the most analytical results. The effective use of these comprehensive analysis results by coaches and athletes is conducive to scientifically tapping the training potential of athletes and improving their sports training results. In the competition stage, by monitoring the competition process and the mental state of the athlete, the status of the athlete is checked, and the competition situation of the athlete is monitored to determine whether the condition adjustment is needed.

When the conjugated material is subjected to external forces, it would cause extremely small changes that are not visible to the naked eye. The strength of the structure in the crystal changes, and the charge carriers concentrate on the movement. This causes a large change in its resistance value, which is reflected in the large change in the resistance value of the resistor made of its components (Seifrid et al., 2019; Fu et al., 2021). Under the action of stress, the relative change of resistance value of conjugated materials is:

$$\frac{∆Q}{Q}=\left(1+2\theta \right)\varepsilon +\frac{∆q}{q}$$ (1)

θ is the Poisson ratio of the material; ε is the axial strain; q is the resistivity.

The relative change in the resistivity of the conjugate material is:

$$\frac{∆q}{q}=\gamma R\theta$$ (2)

Strain sensitivity coefficient of conjugated materials:

$$F=\frac{∆Q}{Q}/\theta =\gamma R$$ (3)

The state parameter identification of sports load is established, and the track of state tracking recognition is obtained as follows:

$$h\left(y\right)=sgn\left(\sum_{i=1}^{m}x_{i}^{*}a\left(y_{i}*y\right)+b^{*}\right)$$ (4)

Among them, $b^{*}=a\left(m\right)-\sum_{i=1}^{m}x_{i}*H$ represents the detection statistic of the amount of physical activity load state detection. At the same time, the high-order statistical distribution sequence of sports load state feature recognition can be obtained:

$$K\left(y_{i},y_{j}\right)=\exp\left(B-\mu {\left\|y_{i}-y_{j}\right\|}^{T}\right)$$ (5)

The output expression of condition monitoring is as follows:

$$\beta \left(y_{i},y_{j}\right)=B-{\left[\mu y_{i}^{T}*y_{j}+r\left(m\right)\right]}^{T}$$ (6)

Therefore, it can realize the analysis of sports load state characteristic parameter spectrum and real-time sports state monitoring, and realize the real-time monitoring and health level testing of high-intensity sports according to the monitoring results.

### 3.3 Application of conjugated materials in heart rate monitoring and respiratory monitoring

When the heart performs contraction exercises, blood flows from the heart to the arteries and veins, and at this time the blood volume in the arteriovenous veins increases, resulting in an increase in hemoglobin and an increase in absorption problems. When the heart dilates, blood flows back into the heart, resulting in a decrease in blood volume in the arterioles and capillaries, as well as a decrease in the amount of hemoglobin and the ability to absorb incoming light. Therefore, when the heart beats regularly, the amount of blood in the arteries also changes with the heartbeat, resulting in a periodic change in the blood’s ability to absorb light. Blood volume pulse wave refers to the change in the time when the blood volume in the artery jumps at will.

The skin surface would reflect the incident light specular and diffuse, and the calculated heart rate is:

$$F_{i}\left(t\right)=L\left(t\right)*\left(X_{s}\left(t\right)+X_{d}\left(t\right)\right)+X_{m}\left(t\right)$$ (7)

$L\left(t\right)$ represents the intensity level of light; $X_{s}\left(t\right)$ and $X_{d}\left(t\right)$ are specular and diffuse components, respectively.

When the human body moves, it would cause the distance between the human body and the light source and the camera to change, thereby changing the reflection component, which can be expressed in detail by the following formula $H_{i}\left(t\right)$ :

$$H_{i}\left(t\right)=L_{0}\left(1+j\left(t\right)\right)*\left(y_{c}*c_{0}+y_{k}*k\left(t\right)+y_{r}*r\left(t\right)\right)+X_{m}\left(t\right)$$ (8)

$y_{c}$ , $y_{k}$ and $y_{r}$ represent the unit color vector reflected by the skin surface, respectively.

Signal strength and signal details differ in all directions, and the mechanism of signal production would make the body feel the manager. The specific expression is as follows:

$$\sum_{i}H_{i}=x*n^{*}=-H_{r}+H_{c}-H_{b1}{-H}_{b2}+H_{b}+H_{q}$$ (9)

$H_{r}$ is the push force generated by ejection; $H_{c}$ is the blood mass of ejection in each heartbeat cycle; $H_{b1}$ is the acceleration of blood flow.

The breathing movement of the body is mainly a process of gas exchange. Breathing can be divided into internal breathing and external breathing, and normal breathing refers to external breathing (Chen et al., 2019; Mu et al., 2019). The introduction of different respiratory monitoring methods is shown in Table 2.

**TABLE 2:** Different respiratory monitoring methods.

Respiratory monitoring method	Category	Classification	Introduction
	Contact type	Respiratory airflow monitoring method	The breathing air flow is collected through the breathing mask, and then the breathing signal is measured by the gas flow meter inside the instrument
		Thermocouple monitoring method	The temperature change is measured by a thermocouple to obtain a breathing signal
		Respiratory induction plethysmography	The breathing signal can be obtained by detecting the size of the magnetic flux
		Impedance monitoring method	The electrical-grade patch is used to measure the impedance changes of the chest cavity (human body), and then the respiratory signal is obtained
	Non-contact	Infrared imaging technology	The parameters of the breathing signal are indirectly obtained through the thermal imaging of the facial area during breathing
		Biological radar technology	Biological radar is used to emit electromagnetic waves to the human body, and the received echo signal is analyzed for micro-motion characteristics to obtain a respiratory signal
		Monitoring method based on video image processing technology	Through image processing technology, the movement displacement of the chest and abdomen during breathing is amplified, and the breathing signal is extracted from it
		Monitoring method based on non-contact microphone	Using a non-contact microphone, the sound of air flow generated during breathing is collected, the sound signal is processed and analyzed, and the breathing state is obtained

There is a clear baseline drift from the original breath signal. In order to obtain obvious respiratory signals, the original signal should first be de-baseline drift processing:

$$f\left(t\right)=x_{0}+x_{1}t+x_{2}t^{2}+\cdots +x_{m}t^{n}=\sum_{k=0}^{n}x_{k}t^{k}$$ (10)

In the formula, n is a positive integer, representing the order of the polynomial.

Considering that the amplitude of the respiratory signal is easily changed with the depth of breathing, the current common threshold method mainly sets the threshold manually:

$$H=\vartheta *\left[\mathrm{Max}\left(m_{1}\right)+\mathrm{Max}\left(m_{2}\right)+\mathrm{Max}\left(m_{3}\right)+\mathrm{Max}\left(m_{4}\right)+\mathrm{Max}\left(m_{5}\right)\right]$$ (11)

### 3.4 Specific applications of conjugated materials in motion monitoring

Conjugated materials have many practical applications in motion monitoring, and conjugated polymer materials can be used to manufacture flexible sensors, such as pressure sensors and telescopic sensors. These sensors can be attached to the skin to monitor joint movement and muscle activity. They can measure pressure, stretching, and deformation, thereby recording and analyzing movement posture, amplitude, and force in real-time. Conjugated materials can also be integrated into textiles to manufacture smart textiles, such as smart sportswear and smart compression socks. These textiles can monitor body movements and physiological indicators, such as heart rate, respiratory rate, step count, etc. They can provide a comfortable wearing experience and transmit data to mobile devices for analysis and recording through wireless connections. Conjugated polymer materials can also be used to manufacture optical sensors and play a role in motion monitoring. For example, fluorescent materials can be used to label certain compounds in the body (such as oxygen, pH value, etc.) to evaluate the physiological state of the body during exercise. These fluorescent sensors can monitor changes in indicators in real-time, providing insights into physiological responses during exercise. Conjugated materials can also be used to construct biosensors for detecting specific biological molecules or indicators. For example, conjugated polymers can be modified on the surface of electrodes and used in exercise monitoring to detect indicators such as lactate concentration and glucose concentration in the blood, thereby evaluating energy metabolism and body fatigue during exercise. In addition, conjugated materials can also be used to manufacture micro sensors and electronic devices for recording and storing motion data. These devices can be built into items such as sports devices, shoes, or watches, and analyze motion patterns, techniques, and effects by collecting data such as acceleration, posture, and angle. From the above examples, it can be seen that conjugated materials provide more in-depth exercise data and detailed analysis for athletes, helping them optimize exercise training, improve body posture, and prevent injuries.

## 4 Application of conjugated materials in the improvement of sports equipment

### 4.1 Application of conjugated materials in sportswear

#### 4.1.1 Research on elements of color design

**FIGURE 4:**
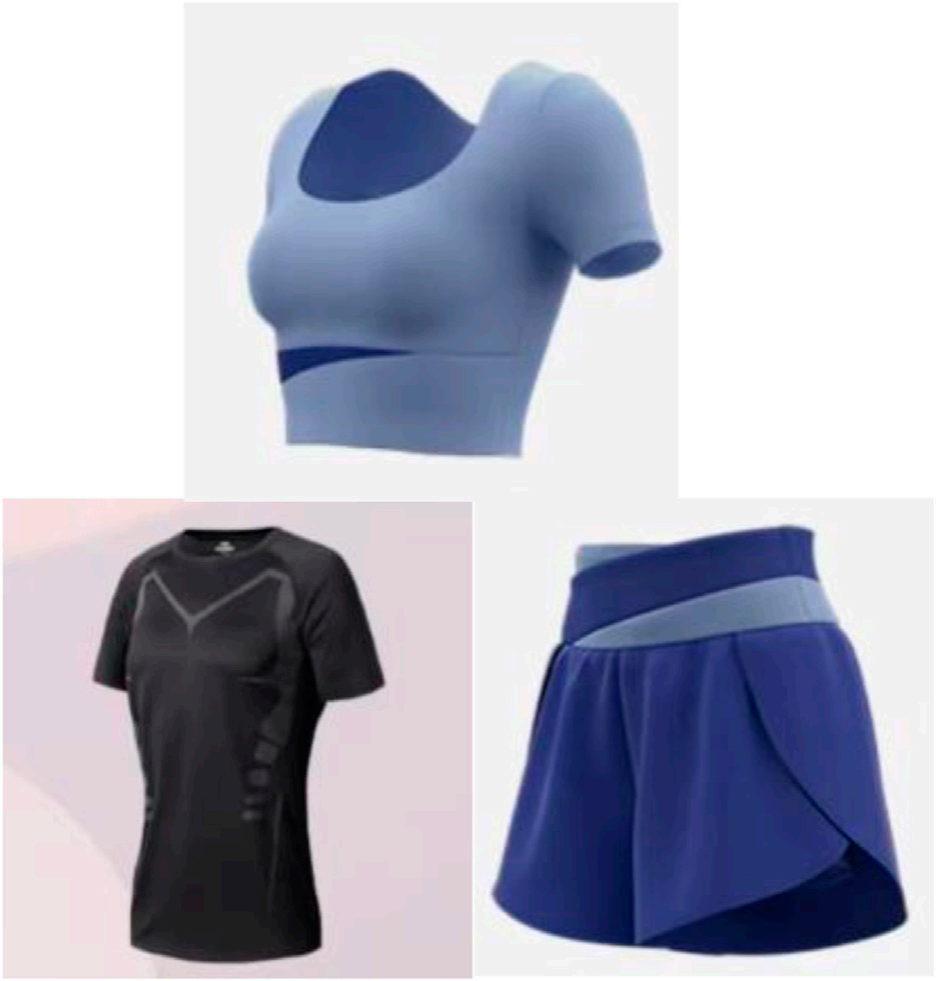
Color design of sports training clothing based on conjugated materials.

In the use of seamless knitting process, conjugated materials can be used to design sports clothing colors. The design rules are generally the same as those for other materials (Hu et al., 2019; Hassabo et al., 2023). Most of them are monochrome design, with black, blue, red and other main colors, followed by different color design, and a small amount of color design is completed by printing. In the past, the overall tone of sports training clothing should present a beautiful and vibrant feeling, and the colors would mostly be high brightness and high purity bright colors. However, with the popularity of sportswear styles and the continuous development of conjugated materials, by applying conjugated materials to the design of sportswear colors, designers can carry out different innovative designs according to different preferences, design characteristics and the style he wants to express. The color design of sports training clothing based on conjugated materials is shown in Figure 4.

#### 4.1.2 Waterproof and moisture permeability requirements

**FIGURE 5:**
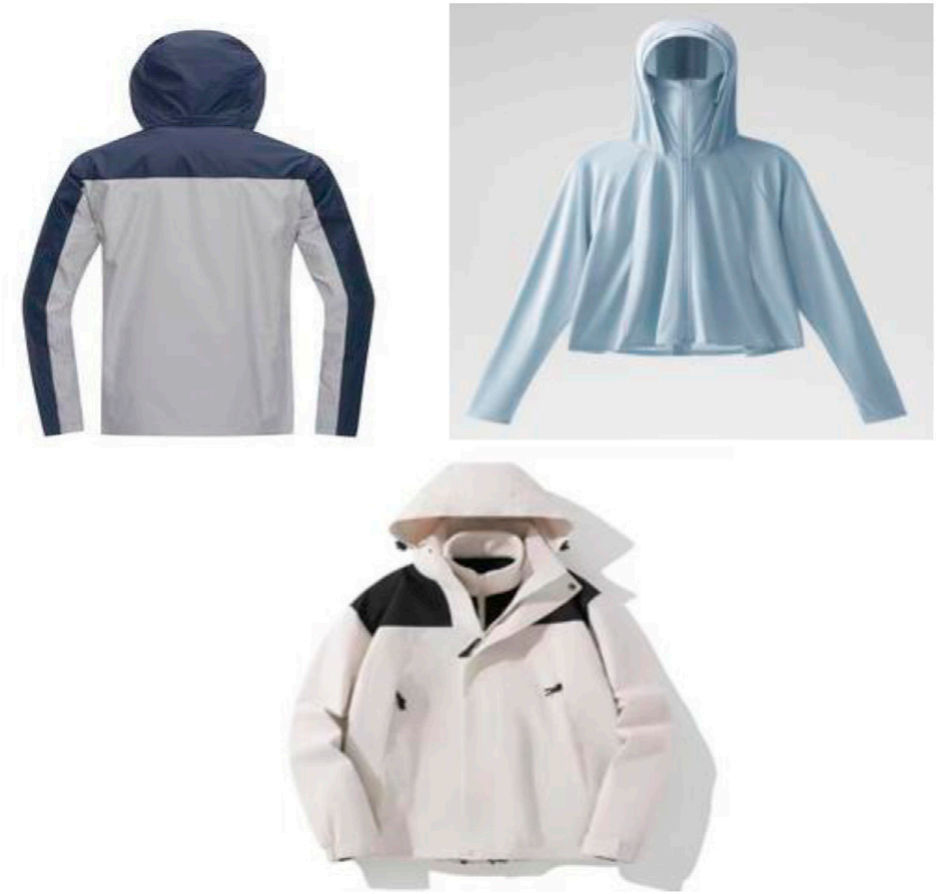
Waterproof sportswear design based on conjugated materials.

If people stop to rest after a lot of exercise, the temperature outside is too low, and the sweat can not be dispersed in time, it would lead to the formation of water droplets on the inner layer of clothes, making people feel uncomfortable, which is the so-called “dew” phenomenon. The comfort of sports clothing requires that clothes not only prevent rain and snow from getting wet, but also release sweat in the body in time. The sweat of the human body is divided into visible sweat and invisible sweat. During the moisturizing process of clothing, both gaseous and liquid moisture changes. Invisible sweat refers to water vapor that evaporates from the surface of the human skin, that is gaseous water. Visible sweat, also known as sweat, is the sweat produced when the human body sweats more or when the temperature is too high. Due to the special wear purpose of motion, its fluid transfer performance is particularly important. Conjugated materials have advantages in waterproof and breathable. The use of its clothing, it has a strong moisture permeability, in order to facilitate the timely dissemination of sweat, promote the human body’s heat and humidity balance can be full of the following conditions: 1) sweat can be quickly core absorption or migration to the outer surface of the clothing, that is, has a good sweating effect. 2) If there is sweat left in a short time, it can prevent sweat flow. 3) After the fabric moisture absorption, it can be quickly moistened and dried. In addition, based on the conjugated materials made of clothing, clothing weight is reasonable, light load. This allows for unhindered air circulation in the garment, so that the heat balance of the torso can be achieved. The structure of waterproof sportswear based on composite material is shown in Figure 5.

### 4.2 Application of conjugated materials in sports footwear products

#### 4.2.1 Reduce the thickness of the sole

The use of conjugated materials can make a thin thickness of the sole, reduce the thickness of the sole, and can effectively achieve the purpose of lightweight sole measurement. The thickness of the sole includes the thickness of the sample and the thickness of the bottom plate, usually just reducing the thickness of the bottom plate. The reduction of the bottom plate itself does not require the support of high-tech equipment, but when the thickness of the sole is reduced to a certain extent, it must be supplemented by corresponding measures, and the use of conjugated materials can increase the wear resistance and high tensile strength of the sole, thereby increasing the performance of the sole.

#### 4.2.2 Change the structure of the sole

**FIGURE 6:**
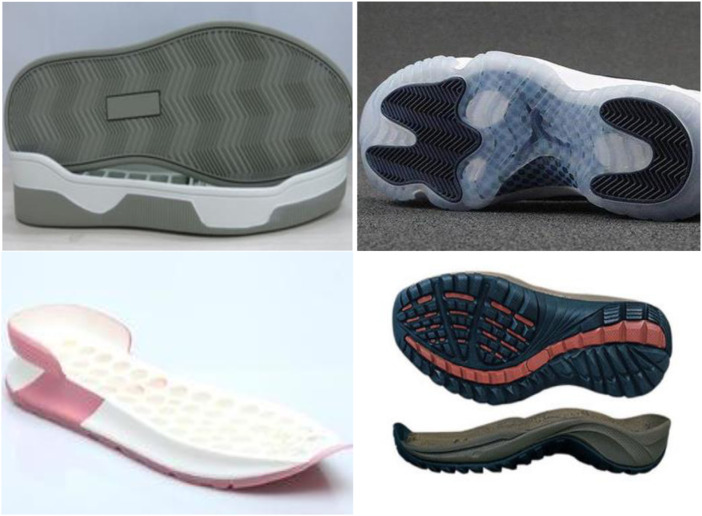
Sole design of sports training shoes based on conjugated materials.

Early sneakers were usually boat-like socks with side walls, often with a mesh pattern on the inside of the bottom, making the entire sole look thick and large. With the continuous development of shoemaking technology, materials and processes, conjugated materials are applied to the production of sports shoes, and the running shoe body has begun to become more beautiful. Conjugated materials can be used to replace part or all of the side wall of the sole, and removing or reducing the mesh structure of the sole would reduce the quality of the sole and change the appearance and structure of the sole. The structure of football shoe soles based on connection information is shown in Figure 6.

### 4.3 Application of conjugated materials in sports equipment

Ball sports equipment usually includes tennis rackets and badminton rackets, which usually include two parts: a handle and a racket face. The handle is the part that the player must hold with his hand when playing, so the material of the handle must have good friction to prevent the player from loosening the racket during the force process. At the same time, the material of the handle should have high strength and light texture, and high strength is conducive to the athlete’s not breaking during the exercise. Light weight is conducive to reducing the physical loss of athletes during the game, and conjugated materials perfectly fit these two points. For sports equipment, surface material is also important (Midha and Rahela, 2018; Li and Wang, 2022). The main function of the racket head is to hit the tennis ball and the tennis ball in motion, and the racket head should be well controlled in the movement of the tennis ball. Therefore, the racket surface should have good impact, shock resistance and ductility, and conjugated materials just have this characteristic. When the racket face hits the moving ball, vibration is generated, and reducing the vibration of the racket face would help the player correctly judge the coming of the ball and achieve control of the ball path through the necessary force and control. Conjugated materials have good shock absorption properties, and can meet the needs of the equipment on the handle and the surface of the racket. Therefore, conjugated materials have great applications in the production of ball sports equipment.

## 5 Related applications of conjugated materials in sports training

During sports training, the clothes and shoes worn are very important for the safety and assistance of sports training. Sports clothing and shoes made of good materials can be of great help to athletes during sports training. Conjugated materials have the characteristics of light material, strong water resistance, and strong breathability. Using them for the production of sports clothes and shoes, the user’s experience is better. In order to further demonstrate that it can bring users better sports, this paper randomly selected 20 sports players and asked them to rate the clothes and shoes made of conjugated materials. It is mainly scored from six aspects such as comfort, water resistance, portability, lightness, beautiful and breathability, and the scoring score is 10 points. The higher the score, the higher the satisfaction of the sports participants. The specific score results are shown in Table 3.

**TABLE 3:** The scores of sports participants on garments made based on conjugated materials.

Classification	Clothes						Shoes					
Number Indicator	Comfort	Water resistance	Portability	Lightness	Aesthetics	Breathability	Comfort	Water resistance	Portable	Lightness	Aesthetics	Breathability
**1**	9.2	8.9	9.05	9	9	9.2	9	8.9	9	9.1	9	9.1
**2**	9.1	8.9	9.2	9.1	9	9.1	9	9	9	9.1	9	9
**3**	9	9	9.15	9	9	9.1	9.1	9	9	9.1	9.1	9
**4**	9	9	9	9	9	8.9	9.1	9.3	9	9.1	9.1	9.05
**5**	8.8	9.05	9	8.8	9	8.95	9	9.1	9.2	9	8.7	9.05
**6**	8.85	9.05	9.1	9.1	9	8.95	9	9.1	9.15	9.05	9	9.2
**7**	8.9	9.2	9	9.1	9	9	9.05	9	9	9.05	8.8	8.95
**8**	9	9.15	9	8.9	9	9	9.05	9	9	9.05	9.1	9.1
**9**	9	9	8.8	9	9.1	9.05	9.2	9	8.8	9.1	9.1	9.1
**10**	9.3	9	8.7	9	9.1	9	8.95	9.1	9.1	9.2	8.9	9
**11**	9.1	9	8.9	9	9	9	8.9	9.1	9.1	9.1	9	9
**12**	9.1	8.85	8.9	9.1	9	9	8.95	8.95	8.9	9.1	9	8.9
**13**	8.9	9	9	9.1	9.2	9.05	9	8.95	9	9.1	9.2	9
**14**	8.95	8.9	9	8.9	8.95	9.1	9	9	9	9.1	9.2	9
**15**	9	8.95	9.1	9	9	9.1	9	9	9	9	9.1	8.9
**16**	9.1	9	9.1	9	9.1	9	9	9	9.2	9.05	8.95	8.9
**17**	9.2	9	9.2	9.2	9	9	9	9.2	9	9.1	9	8.9
**18**	9.2	9	9	9.2	9	9.1	9.1	9.3	8.95	9.2	9	9
**19**	9.1	9	9	9	9	9.05	9.1	9.1	9	9.1	9	9
**20**	9.15	9.2	9	9	9.2	9.15	9.2	9	9	9	9.1	9.2

**FIGURE 7:**
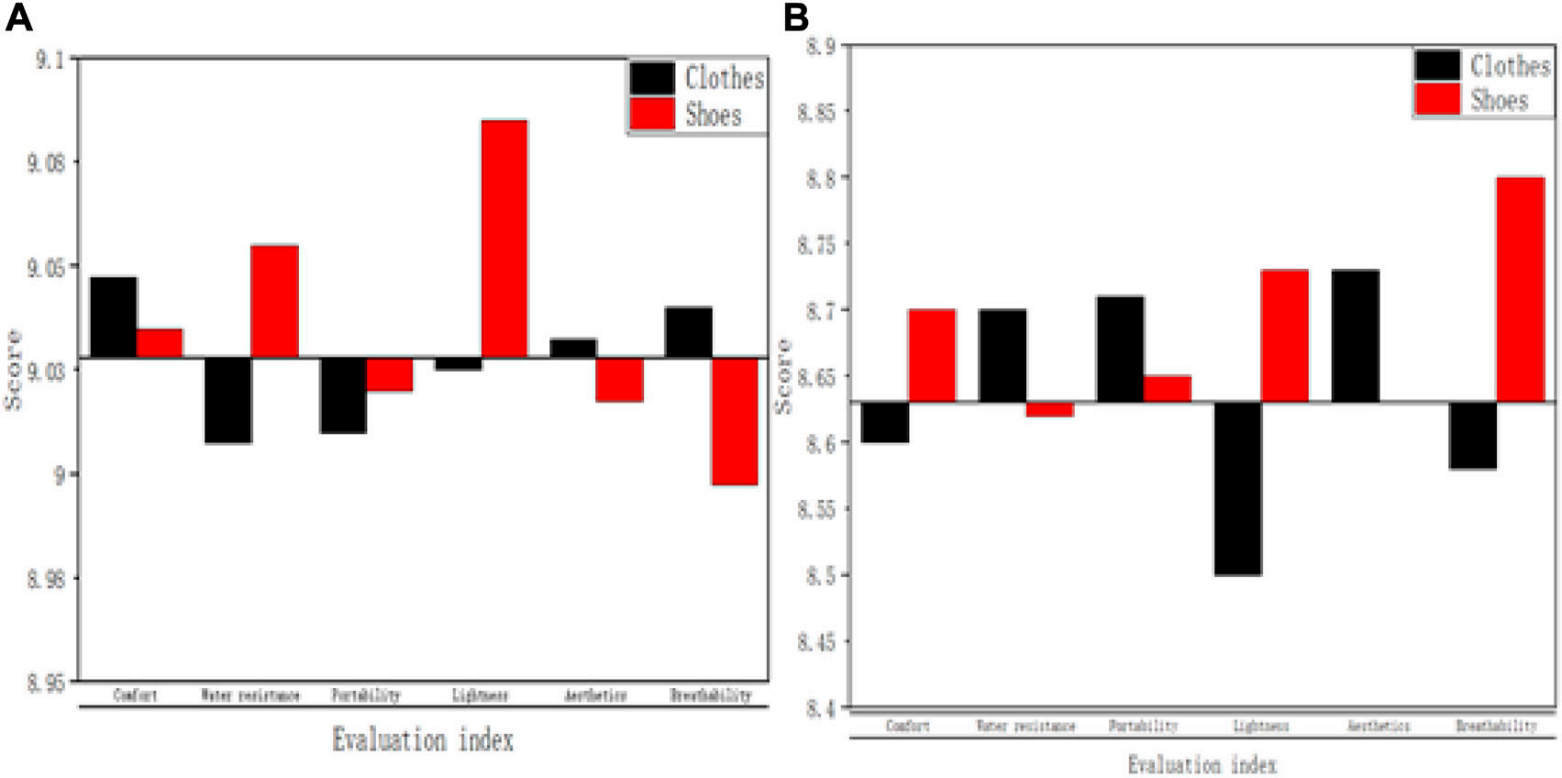
Comparison of the mean scores of sports participants for the two materials. **(A)** Conjugated materials. **(B)** New chemical materials.

As shown in Table 3, 20 athletes were selected, and they were surveyed to wear clothes and shoes made of conjugated materials. After a period of fitting, this paper conducts a score of satisfaction. It can be found that for clothes, the scores of the six aspects of comfort, water resistance, portability, lightness, aesthetics and breathability were above 8.79 points, 8.84 points, 8.69 points, 8.79 points, 8.94 points and 8.89 points. For shoes, the score of comfort, waterproof, portability, lightness, aesthetics and breathability was above 8.89 points, 8.89 points, 8.79 points, 8.99 points, 8.69 points and 8.89 points. The scores are all very high, which indicates that sports athletes are more satisfied with clothes and shoes made of conjugated materials, and also indicates that conjugated materials can improve the breathability and comfort of sports clothing. This can help the sports players to better carry out sports training and achieve more satisfactory results. In this paper, the data obtained in Table 3 are compared with sports clothes and shoes based on new chemical materials. It mainly compares 20 athletes. For clothes and shoes, the average score of the six aspects of appeal is shown in Figure 7.

In Figure 7A and 7B, the specific evaluation indexes of *x*-axis are respectively comfort, water resistance, portability, lightness, aesthetics and breathability. The *Y*-axis is the score of the sports participants. In Figure 7A, the line in the figure was positioned at 9.035. In Figure 7B, the line in the diagram was positioned at 8.63. As shown in Figure 7, it can be found that the sports participants rated the clothes and shoes made based on conjugated materials better, and the evaluation effect was better. As shown in Figure 7A, the average scores of 20 sports participants on clothes were 9.0475, 9.0075, 9.01, 9.025, 9.0325 and 9.04 respectively. The average scores for shoes were 9.035, 9.055, 9.02, 9.085, 9.0175 and 8.9975, respectively. According to Figure 7A, it can be clearly found that based on clothes and shoes made of conjugated materials, sports participants have the largest difference in the average score of lightness, and the smallest difference in the average score of portability. In Figure 7B, the average scores of 20 sports participants on clothes were 8.6, 8.7, 8.71, 8.5, 8.73 and 8.58 respectively. It was 0.4475 points, 0.3075 points, 0.3 points, 0.525 points, 0.3025 points and 0.46 points lower than the average value of clothes made based on conjugated materials in these six aspects. The average scores for shoes were 8.7, 8.62, 8.65, 8.73, 8.63 and 8.8, respectively. It was 0.335 points, 0.435 points, 0.37 points, 0.355 points, 0.3875 points and 0.1975 points lower than the average value of shoes made based on conjugated materials in these six aspects.

**FIGURE 8:**
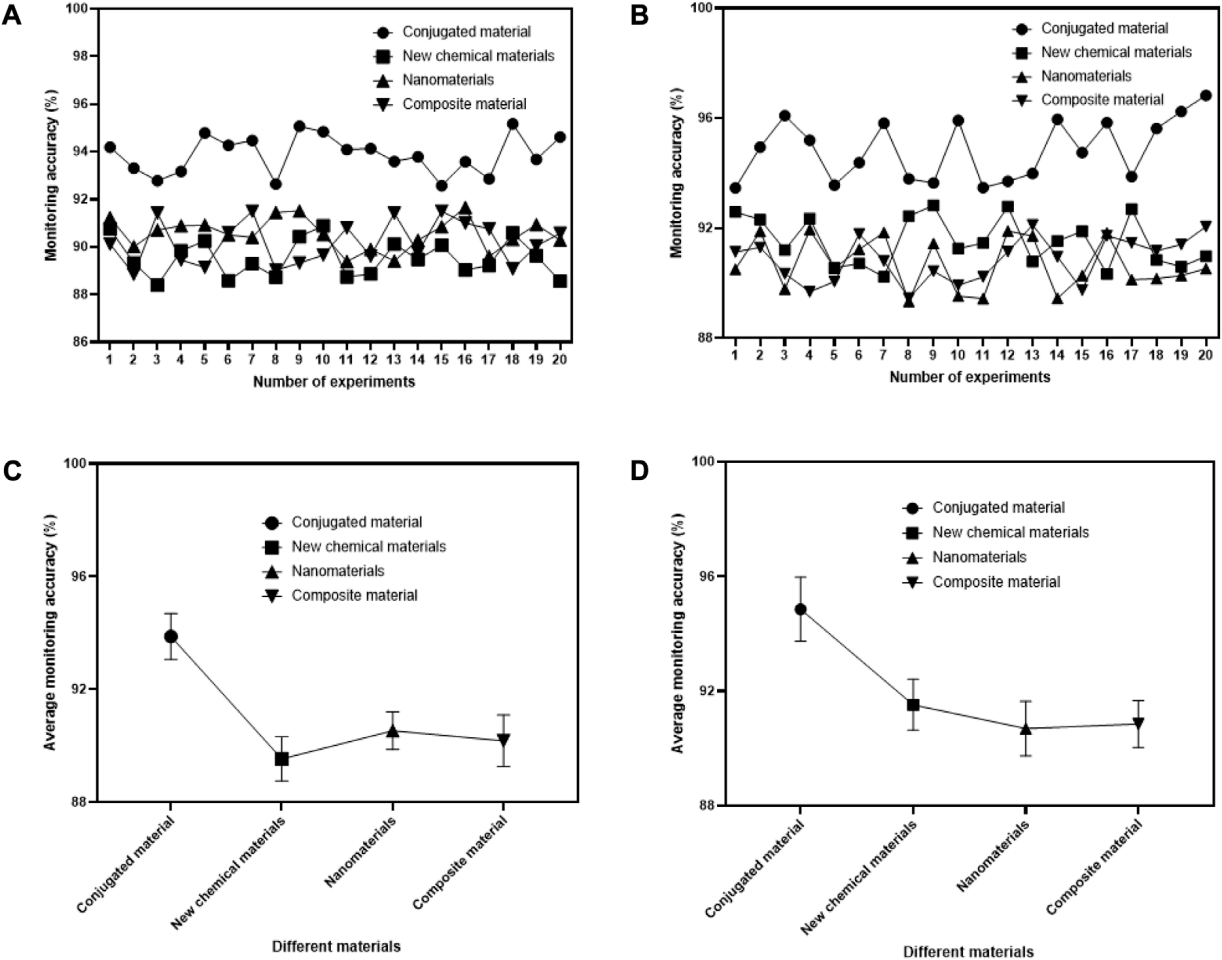
Comparison of monitoring accuracy of different monitoring equipment for sports training. **(A)** Different devices for heart rate monitoring. **(B)** Different devices for respiratory monitoring. **(C)** Mean for heart rate monitoring. **(D)** Mean values for respiratory monitoring.

This article utilizes sensor technology and conjugated materials to monitor the movement status of athletes. By using conjugated materials to construct monitoring equipment, the accuracy of exercise monitoring has been greatly improved, mainly for monitoring heart rate and respiration of athletes. This article conducted 20 tests on the obtained data and compared the monitoring accuracy results with the experimental results obtained by monitoring equipment based on traditional materials, such as chemical new materials, nanomaterials, and composite materials. The specific comparison results are shown in Figure 8.

In Figures 8A, B the *X*-axis represents the number of experiments conducted, with a total of 20 experiments conducted. The *Y*-axis represents the accuracy of monitoring. In Figures 8C, D the *X*-axis represents the monitoring equipment under different materials, and the *Y*-axis represents the average of 20 experimental monitoring. In Figure 8A, the athletes’ heart rate was mainly monitored. It can be found that the accuracy of monitoring equipment based on conjugated materials was much higher than that of other types of monitoring equipment, and the accuracy of monitoring was more than 92.56%. The accuracy of monitoring equipment based on new chemical materials, nanomaterials and composite materials for athletes’ heart rate monitoring was less than 90.9%, 91.67% and 91.51%, respectively. The lowest accuracy of the device based on conjugated materials for heart rate monitoring was only 92.57% in 15 experiments, but it was still 2.49%, 1.72% and 1.07% higher than that of the device based on new chemical materials, nanomaterials and composite materials, respectively. In Figure 8B, the breathing of athletes was mainly monitored. It can be found that the accuracy of monitoring equipment based on conjugated materials was much higher than that of other types of monitoring equipment, and the accuracy of monitoring was more than 93.46%. The accuracy of the monitoring equipment based on the production of new chemical materials, nanomaterials and composite materials for athletes’ breathing monitoring was below 92.84%, 91.96% and 92.13%, respectively. The highest accuracy of the device based on conjugated materials for respiration monitoring was 96.83% in 20 experiments, which was 5.85%, 6.3% and 4.77% higher than that of the device based on new chemical materials, nanomaterials and composite materials, respectively.

As shown in Figure 8C, after 20 experiments, the mean value of heart rate monitoring accuracy of the device built based on conjugated materials was much higher than that of the monitoring device made of other materials, with an average value of 93.8795%. Under 20 experiments of monitoring equipment based on new chemical materials, nanomaterials and composite materials, the average accuracy of athletes’ heart rate monitoring was 89.538%, 90.5385% and 90.181%, respectively. The monitoring accuracy was 4.3415%, 3.341% and 3.6985% lower than that of the device based on conjugated materials, respectively. As shown in Figure 8D, after 20 experiments, the average accuracy of respiratory monitoring devices constructed based on conjugated materials was much higher than that of monitoring devices made of other materials, with an average value of 94.8595%. Under 20 experiments of monitoring equipment based on new chemical materials, nanomaterials and composite materials, the average accuracy of athletes’ breathing monitoring was 91.522%, 90.6905% and 90.851%, respectively. The monitoring accuracy was 3.3375%, 4.169% and 4.0085% lower than that of the device based on conjugated materials, respectively.

Different types of sports equipment are used in sports training. The production of these equipment can use different materials, and the equipment made of different materials brings different feelings to the sports players. Because the conjugated material has the characteristics of light texture, strong durability, good safety and outstanding mechanical properties. Therefore, different types of sports equipment based on conjugated materials can bring sports players better training experience and improve the training effect of sports players. In this paper, 100 sports players are selected to investigate the satisfaction rate of sports equipment made based on conjugated materials such as ball, board, bicycle, racket and pedal. The evaluation indexes are mainly the durability, safety and mechanical properties of sports equipment. In this paper, the satisfaction rate obtained was compared with the sports luck equipment based on new chemical materials, nanomaterials, composite materials and carbon fiber materials. The specific comparison results are shown in Table 4.

**TABLE 4:** The satisfaction rate of sports players to sports training equipment made of different materials.

Classification of different materials	Sports equipment				
	Clubs (%)	Board class (%)	Bicycle class (%)	Racket class (%)	Pedal class (%)
Conjugated material	92	93	94	92	95
New chemical materials	89	85	88	86	90
Nanomaterials	87	86	84	88	88
Composite material	86	86	86	87	89
Carbon fiber material	84	88	87	87	90

As shown in Table 4, for the selected sports participants, the satisfaction rate of different types of sports equipment made of conjugated materials is much higher than that of other sports equipment made of different materials. Among them, the satisfaction rate of sports players based on the equipment made of conjugated materials was more than 91.99%. Based on new chemical materials, nanomaterials, composite materials and carbon fiber materials made of equipment sports satisfaction rate of 90.01% below, 88.01% below, 89.01% below and 90.01% below. The satisfaction rate of sports players for pedal sports equipment based on conjugated materials was the highest, 95%, 5%, 7%, 6% and 5% higher than that based on new chemical materials, nanomaterials, composite materials and carbon fiber materials. The lowest satisfaction rate was the clubs and rackets, both of which were only 92%, the sports equipment of the clubs was 3%, 5%, 6% and 8% higher than the equipment made of new chemical materials, nanomaterials, composite materials and carbon fiber materials, respectively; the sports equipment of the rackets was 6%, 4%, 5% and 5% higher, respectively.

In summary, the main role of conjugated materials in sports training data collection is mainly reflected in their mechanical performance optimization, biocompatibility, chemical stability, photoelectric performance, and thermal stability. Through the conjugation design of materials, different levels and scales of material function regulation can be achieved to adapt to various sports training needs. For example, conjugate design of elastic materials can improve their strength while ensuring elasticity, thereby better absorbing and dispersing the impact force during sports training, and reducing the risk of sports injuries. Partial conjugated materials can also have good biocompatibility with human cells and tissues, which allows them to be used as part of implantable biosensors or sports training monitoring devices to achieve more direct and in-depth monitoring of body condition. In addition, conjugated materials have good chemical stability and can resist the corrosion and erosion of various chemicals. Therefore, in sports training, they can be used to manufacture outdoor sports equipment, such as shoe soles, clothing, etc., to improve the durability and service life of these equipment. Some conjugated materials also have excellent optoelectronic properties, such as conductivity and optical transparency, which enable them to be used in the manufacturing of various wearable electronic devices, such as heart rate monitors, pedometers, etc., to achieve real-time monitoring of various physiological parameters for exercise training. At the same time, conjugated materials usually have excellent thermal stability, which can maintain their structure and performance stability in extreme temperatures and harsh environments. Therefore, in certain extreme sports training scenarios, such as polar exploration and mountain climbing, they can be used to manufacture high-performance sports equipment and protective equipment.

## 6 Conclusion

With the continuous development of the economy and the continuous improvement of people’s lifestyle, people pay more attention to sports and gradually begin to use materials to help better sports training. Conjugated material is a new kind of material. After its appearance, it has rapidly replaced wood materials, nanomaterials and carbon fiber materials in the design of traditional sports equipment with its good performance, especially in the design of high-end sports equipment, and has been widely used. At the same time, because of its excellent optical, electrical, thermal and other properties, it can be applied to experience sports clothing design and sports training monitoring. Through the use of its designed sportswear, with breathability, significantly improved comfort, but also can better monitor the heart rate and breathing of the exercisers during training, at any time to understand the movement of the exercisers, to ensure the safety of the exercise process.

## Data Availability

The original contributions presented in the study are included in the article/supplementary material, further inquiries can be directed to the corresponding author.
